# Efficacy and safety of botulinum toxin type A for treatment of Frey’s syndrome: evidence from 22 published articles

**DOI:** 10.1002/cam4.504

**Published:** 2015-08-26

**Authors:** Shang Xie, Kan Wang, Tao Xu, Xue-Sheng Guo, Xiao-Feng Shan, Zhi-Gang Cai

**Affiliations:** 1 Department of Oral and Maxillofacial Surgery, Peking University School and Hospital of StomatologyBeijing, 100081, China; 2 Department of Stomatology, Capital Medical University Pinggu Teaching HospitalBeijing, 101200, China

**Keywords:** Botulinum toxin type A, efficacy, Frey’s syndrome, proportion meta-analysis, safety

## Abstract

Frey’s syndrome (FS) is an unavoidable sequela following the surgery of the parotid gland. Although several treatment methods are available, their efficacy is short term or accompanied by unacceptable complications. In the past two decades, botulinum toxin type A (BTXA) has been widely used to treat FS. Although several systematic reviews have been reported recently, they were conflicting and with obvious deficiencies. Thus, we performed an objectively systematic review to determine whether BTXA is an effective and safe treatment for FS. A literature retrieval covering PubMed, Web of Science, Ovid, Embase and Cochrane library was performed on 16 January, 2015. Proportion meta-analysis and corresponding 95% confidence interval (CI) were performed to evaluate the efficacy and safety of BXTA in treatment of FS. A total of 499 records were retrieved and 22 articles with 23 studies were included after scrutiny by two independent authors. Statistical analyses regarding the effective rate, incidence of complications were used to estimate the efficacy and safety of BTXA. Our results suggested that the effective rate of BTXA for treatment of FS is 98.5% (95% CI = 0.971–0.994) and the incidence of complication is 3.6% (95% CI = 0.017–0.061). In conclusion, our study supports that BTXA produces meaningful benefits on the treatment of patients with FS. However, owing to lack of strong evidence, future studies with well-designed inclusion criteria and multicenter randomized controlled trials are needed to give more credible evidence, if possible.

## Introduction

Parotid gland tumor is one of the most common neoplasms in the head and neck region, and nearly about 50–100% patients with parotid tumor undergo Frey’s syndrome (FS) after parotidectomy 1–3. Half of them have noticed symptoms and feel the gustatory sweating. About 13–23% patients consider their symptoms troublesome and severe 1,4.

Frey’s syndrome, also named auriculotemporal syndrome or gustatory sweating, is described as facial sweating and flushing during meals following lacerations or surgeries in the region of the parotid gland. Its earliest known description dates back to 1757 by Duphenix 5 and further described by Baillarger 6. However, it was not until 1923 that the Polish neurologist, Lucie Frey, was the first to describe the condition of gustatory sweating and flushing after parotid injury as a new syndrome 7. The mechanism of FS is widely accepted by a named “misdirected nervous regeneration” 8. Injury to the auriculotemporal nerve resulting from parotidectomy or parotid region trauma might damage parasympathetic fibers, sympathetic fibers, and the parotid gland 8. In the process of nerve regeneration, parasympathetic fibers might emerge as a wrong connection and grow along with the cut ends of sympathetic fibers to the sweat glands and skin blood vessels 8,9. Current therapeutic strategies of FS include a variety of drugs and a number of surgical approaches 3,10–13. However, none of them are entirely satisfactory. In 1994, injection of botulinum toxin type A (BTXA) into the hyperhidrotic area had demonstrated the efficacy of reducing or abolishing focal sweating without serious side effects 14,15. Botulinum toxins, produced by the Clostridium botulinum, are a family of neurotoxins including seven subtypes. BTXA is one of the most common subtypes which prevent the release of acetylcholine at nerve terminals and thus blocking neurotransmission. Owing to the “misdirected nervous regeneration” of FS, BTXA has been used successfully to manage the patients with FS since 1995 4,16–18.

To patients and clinicians, any treatment strategies need to be based on good clinical evidence. Otherwise, it might affect the curative effects and even produce seriously unfavorable consequences 19. According to Cochrane Handbook, meta-analysis and systematic review are considered to be the best available evidence if all available trials are included. However, “the best available evidence” might not be equal to “strong evidence” or “sufficient evidence” 20,21. Based on this, it is important to judge the evidence quality to avoid incorrect treatment strategy.

Although BTXA has been widely used for the treatment of FS, its effective rate, incidence of complication, treatment methods, and reinjection effect remains inconsistent. Current inconsistencies about the treatment of BTXA in FS require a systematic review to clarify their potential advantages and disadvantages, which might promote further studies on the topic, and even produce clinical guidelines on the topic in the future.

In this article, we systematically estimate all evidence about treatment methods, reinjection effect, efficacy, and safety of BTXA for treatment of FS to supply evidence for clinical strategy.

## Materials and Methods

### Literature retrieval and selection criteria

A literature retrieval was performed covering the Cochrane library, PubMed, Web of Science, Embase, and Ovid for articles that met the following search strategies: (1) neurotoxin A, botulinum OR clostridium botulinum A toxin OR clostridium BTXA OR botulinum A toxin OR BTXA, and (2) hyperhidrosis, gustatory OR gustatory hyperhidrosis OR gustatory sweating OR auriculotemporal syndrome OR syndrome, auriculotemporal OR baillarger syndrome OR syndrome, baillarger OR von FS OR Syndrome, von Frey’s OR FS OR syndrome, Frey’s OR von Frey syndrome OR syndrome, von Frey OR auriculotemporal nerve syndrome OR Frey syndrome OR syndrome, Frey. In addition, references from retrieved articles were screened to identify possibly missed studies by literature retrieval. All identified studies were screened and judged whether they were eligible for inclusion criteria by two authors independently.

Inclusion criteria: (1) articles were published in English; (2) studies reported clinical outcomes in treatment of FS by BTXA. Exclusion criteria: (1) review, meta-analysis, case report, editorial, meeting abstract; (2) No. of patients is less than 5.

### Data extraction

The key information of all the eligible studies was independently extracted by two authors (Xie and Shan). Inconsistence between two authors was solved by the other authors. The following data were extracted from each eligible study: first author, publication year, country, participants, interventions, controls, outcomes of patient, study design (PICOS), and other relevant information.

### Quality assessment of included studies

Quality assessment of the included studies was performed by two authors dependently according to Centre for Evidence Based Medicine (CEBM): (1a) Systematic reviews of randomized controlled trials (RCTs); (1b) Individual RCT; (2a) Systematic reviews of cohort studies; (2b) Individual cohort study; (3a) Systematic reviews of case-control studies; (3b) Individual case–control study; (4) Case-series, poor quality cohort, poor case control studies; (5) Expert opinion without explicit critical appraisal, or bench research or “first principles”^22^.

### Statistical analysis

Data analysis was performed with STATA 11.0 software (Stata Co., College Station, TX) or StatsDirect software, version 2.8.0 (StatsDirect Ltd, Sale, Cheshire, UK). The risk ratio (RR) or pooled proportion with corresponding 95% confidence interval (CI) was calculated to estimate the effective rate and side-effect incidence of BTXA in treatment of FS. STATA 11.0 software was used if all included studies were presented in a compatible form. Otherwise proportion meta-analysis for this systematic review was compiled by StatsDirect software (version 2.8.0; StatsDirect Ltd). The inconsistency index *I*-squared was used to estimate the variation caused by heterogeneity 23. When *P* > 0.10 and *I*^2^ < 25%, the fixed-effect model was used, indicating that interstudy heterogeneity was not significant. Otherwise, a random effect model was performed.

## Results

### Literatures retrieval and study description

**Figure 1:**
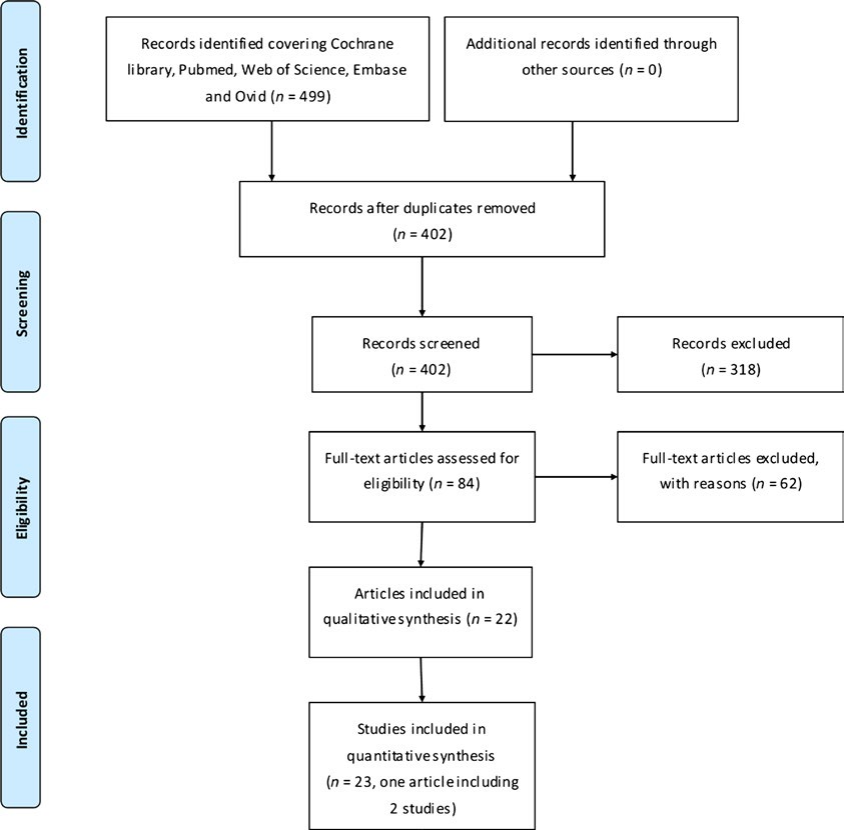
Flow diagram of the literature retrieval.

A total of 499 records were identified though literatures retrieval and 402 articles were left after excluding duplicates. Of which, 318 records were dropped out for unconformity with our issues. The remaining 84 were considered potentially eligible and their full-text articles were screened. After serious scrutiny for eligibility, 22 articles 5,24–44 including 23 studies met our inclusion criteria and were included for our study (Fig.1).

The main information of included studies was summarized in Table1, including first author, publication year, country, participants (number of patients, gender, age), interventions, control, outcomes (efficacy, safety, follow up time), study types and other necessary data. Of these included studies, 411 patients were included to analyze the efficacy and safety of BTXA in treatment of FS (Table1). The follow-up time ranged from 1 to 29 months and the mean duration of effect varied from 3 to 20 months. The botox concentrations were between 16 U/mL and 75 U/mL, and the maximal dose used in per participants was 380 U. The interjection distances between the two injection sites were 10, 15, or 20 mm (Table2).

**Table 1:** Charactristics of included studies

First author, year	Country	Participants	Interventions	Control	Outcomes		Study types	Quality levels
					Efficacy	Safety		
M. Naumann, 1997	Germany	*N* = 45(20 male and 25 female; mean age 52.8 years; patients suffered from disabling FS)	CB: 2.0 U/0.1 mL; V: 0.1 mL; ID: 15 mm; MDU: 72 U	No	Half the patients rated gustatory sweating subjectively as completely abolished, and the remainder felt pronounced improvement; This rating was essentially unchanged 6 months after injection	No side effects except for occasional small cutaneous hematomas (8 patients) or moderate pain at the time of or after injections (10 patients)	Case series	level 4
A. Bjerkhoel, 1997	Sweden	*N* = 14 (age range 20–74; patients had noticed sweating during meals)	CB: 2.5 U/0.1 mL; V: 0.1 mL; ID: 10 mm; MDU: 62.5 U	No	Follow up time: 1–13 months; and only 1 patient was recurrent at 13 months	One patient had a transitory discrete effect on the mimic muscles at the corner of the mouth	Case series	level 4
R. Laskawi, 1998	Germany	*N* = 19 (10 male and 9 female; mean age 52 years; patients with FS)	CB: 2.5 U/0.1 mL; V: 0.1 mL; ID: 20 mm; MDU: 100 U	No	Full effect of the first injection lasted for a period ranging from 11 to 27 months (mean, 17.3 months)	Side effects were absent	Case series	level 4
O. Laccourreye, 1999	France	*N* = 33 (16 male and 17 female, mean age: 48 years; patients with FS)	CB: 2.5 U/0.1 mL; V: 0.1 mL; ID: 10 mm; MDU: 175 U	No	Minimum follow-up time: 18 months. None of variables statistically related to the severity of the recurrent FS and mean duration of effect was 10.2 mol/L for reinjection	Injection pain in 5 patients; Slight and partial weakness of the upper lip and a numbness of the cheek in 2 patients. Recovery within 3 months	Case series	Level 4
J. J. von Lindern, 2000	Germany	*N* = 7 (4 male and 3 female; mean age 42; patients with serious FS)	CB: 2.0 U/0.1 mL; V: 0.1 mL; ID: 20 mm; MDU: 380 U	No	No patient required a reinjection of type A botulinum toxin in the medium or long term observation period (up to 23 months)	No side-effect	Case series	Level 4
P. Dulguerov, 2000	Switzerland	*N* = 15 (Patients with symptomatic FS)	CB: 5.0 U/0.1 mL; V: 0.1 mL; ID: 10 mm; MDU: 75 U	No	Frey syndrome disappeared in all patients, however, the follow-up of our patient population is shorter than 1 year (median, 3 months)	Weak pain of the intradermal injection	Case series	Level 4
A. Arad-Cohen, 2000	America	*N* = 7 (4 male and 3 female, patients with serious FS)	CB: 2.5 U/0.1 mL; V: 0.1 mL; ID: 10 mm; MDU: 30 U	No	All patients were free of symptoms 5 to 24 months and mean duration of effect was 12.1 months	Adverse effects included a temporary slight weakness of the upper lip in 1 patient	Case series	Level 4
S. Rodopoulou, 2001	Greece	*N* = 9 (5 male and 4 female, ranging in age from 30 to 71 years; patients with noticed FS)	CB: 2.5 U/0.1 mL; V: 0.1 mL; ID: 15 mm; MDU: 34 U	No	At the time of writing (14 months post-injection) all patients were free of symptoms except one had a slightly positive Minor’s test	Without any side effects or complications	Case series	Level 4
R. Laskawi, 2001	Germany	*N* = 43 (23 males and 20 females, mean age 55 years, patients with unacceptable FS)	CB: 2.5 U/0.1 mL; V: 0.1 mL; ID: 20 mm; MDU: NA	No	All patients with gustatory sweating treated with botulinum toxin A finally showed subjective and objective complete cessation of pathological sweating	No severe side effect. 5 patients suffered from small cutaneous hematomas, moderate pain and redness of the skin at the time of or shortly after injections	Case series	Level 4
V. Tugnoli, 2002	Italy	*N* = 17 (9 males and 8 females, aged from 20 to 80 years; patients with severe FS)	CB: 2.5 U/0.1 mL; V: 0.1 mL; ID: 15 mm; MDU: 55 U	No	The clinical effect lasted 7–18 months in 6 patients, while both symptoms were still absent 9–18 months after the BoNT injections in 11 patients	No adverse effects	Case series	Level 4
O. Guntinas-Lichius, 2002	Germany	*N* = 20 (13 male and 7 female; Mean age 53 years)	CB: 2.5 U/0.1 mL; V: 0.1 mL; ID: 10 mm; MDU: 37 U	No	Mean duration of effect were 8.3 months	Adverse effects were not encountered	Case series	Level 4
O. Guntinas-Lichius, 2002	Germany	*N* = 20 (11 male and 9 female; Mean age 47 years)	CB: 5.0 U/0.1 mL; V: 0.1 mL; ID: 10 mm; MDU: 62 U	No	Mean duration of effect were 16.5 months	Adverse effects were not encountered	Case series	Level 4
A. J. F. Beeren, 2002	Netherlands	*N* = 13 (6 male and 7 female, mean age 44, all patients with FS)	CB: 7.5 U/0.1 mL; V: 0.1 mL; ID: 20 mm; MDU: 150	No	Mean recurrence time is 11 months (range 3-24); the recurrent patients were given a second treatment; and the first recurrences were seen only after 15 months	Two temporary perioral muscle paresis; recovery compete within 12 weeks	Case series	Level 4
A. Eckardt, 2003	Germany	*N* = 33 (13 male and 20 female, mean 42 years, patients with FS)	CB: 2.25 U/0.1 mL; V: 0.1 mL; ID: 15 mm; MDU: 80 U	No	Follow up 6–18 month, in 9 patients (27.6%) partial recurrence was seen after 12 months	No complications were reported	Case series	Level 4
D. E. Kyrmizakis, 2004	Greece	*N* = 11 (5 male and 6 female, age range 29–78)	CB: 2.5 U/0.1 mL; V: 0.1 mL; ID: 20 mm; MDU: 52.5 U	No	Follow-up time ranged 6–23 months and one patient had recurrence after 16 months and was retreated successfully	Not reported	Case series	Level 4
C. C. Wang, 2005	Taiwan, China	*N* = 10 (4 male and 6 female; mean age 49 years, patients with serious FS)	CB: 2.5 U/0.1 mL; V: 0.1 mL; ID: 10 mm; MDU: 137.5 U	No	Effective duration for these 13 injections ranged from 2 to 28 months (mean, 9.3 ± 8.1 months)	No adverse effects	Case series	Level 4
M. Pornprasit, 2007	Thailand	*N* = 9 (3 male and 6 female, mean age 45 years; patients with noticed FS)	CB: 2.0 U/0.1 mL; V: 0.1 mL; ID: 10 mm; MDU: 32 U	No	All of the patients showed improvement after 4–7 days.systme-free 9.2 months (7–10)	Dry mouth in 3 patients and disappeared in 1–2 weeks	Case series	Level 4
K. Luna Ortiz, 2007	México	*N* = 23 (8 male and 15 female)	CB: 2.5 U/0.1 mL; V: NA; ID: NA; MDU: 42 U	No	3 patients no response was obtained at 3 months and the application of an additional dose of botox produced no response in 2 of 3 after 6 months	Not reported	Case series	Level 4
D. M. Hartl, 2008	France	*N* = 17 (4 males and 13 females, patients with FS)	CB: 2.5 U/0.1 mL; V: 0.1 mL; ID: 10 mm; MDU: 125 U	No	Patients were free of gustatory sweating for a median duration of approximately 1.5 years	Only one patient had a significant side effect: transient (4 weeks) paresis of the orbicularis oris muscle. Seven patients (41%) found the injections painful	Case series	Level 4
P. M. Diaz, 2008	Spain	*N* = 10 (6 male and 4 female, range 34–70, patients with FS)	CB: 1.6 U/0.1 mL; V: 0.1 mL; ID: 10 mm; MDU: 84 U	No	Patients reported having suffered from a repeat of annoying symptoms an average of 15 months after the first injection	Side effects: dry mouth in two patients and slight muscular weakness while chewing in one patient	Case series	Level 4
P. Capaccio, 2008	Italy	*N* = 6 (2 male and 4 female, age range 26-76)	CB: NA; V: NA; ID: 10 mm; MDU: 100 U	No	Follow up time 4-6 months, clinical recovery	No major side effects were reported	Case series	Level 4
R. de Bree, 2009	Netherlands	*N* = 22 (12 male and 10 female, mean age 51 years; All patients underwent a formal superficial parotidectomy treatment)	CB: 1.875 U/0.1 mL; V: 0.1 mL; ID: 20 mm; MDU: 300	No	The mean (SD) interval was 5.2(3.6) months between the 1st and 2nd treatments (n = 22), 9.6(10.3) months between the 2nd and 3rd treatment, 15.1 months between the 3rd and 4th treatments, 9.3 months between 4th and fifth treatments, 29 months between fifth and sixth treatment, and 25 months between the sixth and seventh treatments	The treatment was well tolerated by all patients and complications did not occur	Case series	Level 4
A. Steffen, 2012	Germany	*N* = 8 (4 male and 4 female. serious Frey’s syndrome)	CB: 5.0 U/0.1 mL; V: 0.05 mL; ID: 10 mm; MDU: NA	No	Mean duration of effect: 7.75 months; range 3–14 months	Not reported	Case series	Level 4

CB, concentration of Botox; V, volume per injection site; ID, interjection distance; MDU, maximal dose used; FS, Frey’s syndrome; NA, not available.

**Table 2:** Original data for BTXA in treatment of frey’s syndrome

First author	Year	No. of participants	CB (U/mL)	*V* (mL)	ID (mm)	Total dose per patient (U)	Mean duration of effect (M)	Note
M. Naumann	1997	45	20	0.05–0.1	15	21.1 (5–72)	>6	
A. Bjerkhoel	1997	14	25	0.1	10	37.7 (17–62.5)	>6.6	2
R. Laskawi	1998	19	25	0.1	20	31.3 (2.5–100)	17.3	2
O. Laccourreye	1999	33	25	0.1	10	86 (25–175)	10	2
J. J. von Lindern	2000	7	20	0.1	20	297 (250–380)	>20	
P. Dulguerov	2000	15	50	0.1	10	15–75	>3	
A. Arad-Cohen	2000	7	25	0.1	10	22.2 (12.5–30)	12.1	2
S. Rodopoulou	2001	9	25	0.1	15	22.66 (12.5–34)	>14	
R. Laskawi	2001	43	25	0.1	20	NA	NA	2
V. Tugnoli	2002	17	20	0.1	15	25–55	>7 (7–18)	2
O. Guntinas-Lichius	2002	20	25^1^	0.1	10	About 37	8.3	2
O. Guntinas-Lichius	2002	20	50^1^	0.1	10	About 62	18.5	2
A. J. F Beeren	2002	13	75	0.1	20	100 (67.5–150)	11	2
A. Eckardt	2003	33	22.5	0.1	15	16–80	>12	2
D. E. Kyrmizakis	2004	11	25	0.1	20	22.5 (15–52.5)	10	2
Chen-Chi Wang	2005	10	25	0.1	10	46.4 (15–137.5)	9.3 (2–28)	2
M. Pornprasit	2007	9	20	0.1	10	10.6 (2–32)	9.2	
Kuauhyama Luna Ortiz	2007	23	25	NA	NA	0.71–42	About 6	
D. M. Hartl	2008	17	25	0.1	10	96 (55–125)	18	
P. M. Diaz	2008	10	16	0.1	10	38 (17–84)	15	2
P. Capaccio	2008	6	NA	NA	10	41 (25–100)	>4	2
R. de Bree	2009	22	18.75^1^	0.1	20	101 (30–300)	5.2	2
A. Steffen	2012	8	50^1^	0.05	10	Unavailable	7.8	

CB, concentration of Botox; V, volume per injection site; ID, interjection distance; MDU, maximal dose used; FS, Frey’s syndrome; NA, not available.

1 2.5 U botox = 10 U disport.

2 Reinjection for recurrent patients without any complications.

### Results of quality assessment

All included studies were case series. According to the CEBM assessment system, the quality of the evidence was level 4 (The results were shown in Table1). Obviously, this estimation system of CEBM may help in analyzing objectively the published literatures and making reasonable clinical decisions.

### Proportion meta-analysis

Owing to the original data presented in an incompatible form, proportion meta-analysis was performed to calculate the pooled proportion and corresponding 95% CI. Statistical analyses regarding effective rate and incidence of complications were calculated and were used to systematically estimate the efficacy and safety of BTXA.

### Current evidence on the efficacy of BTXA in treatment of FS

**Figure 2:**
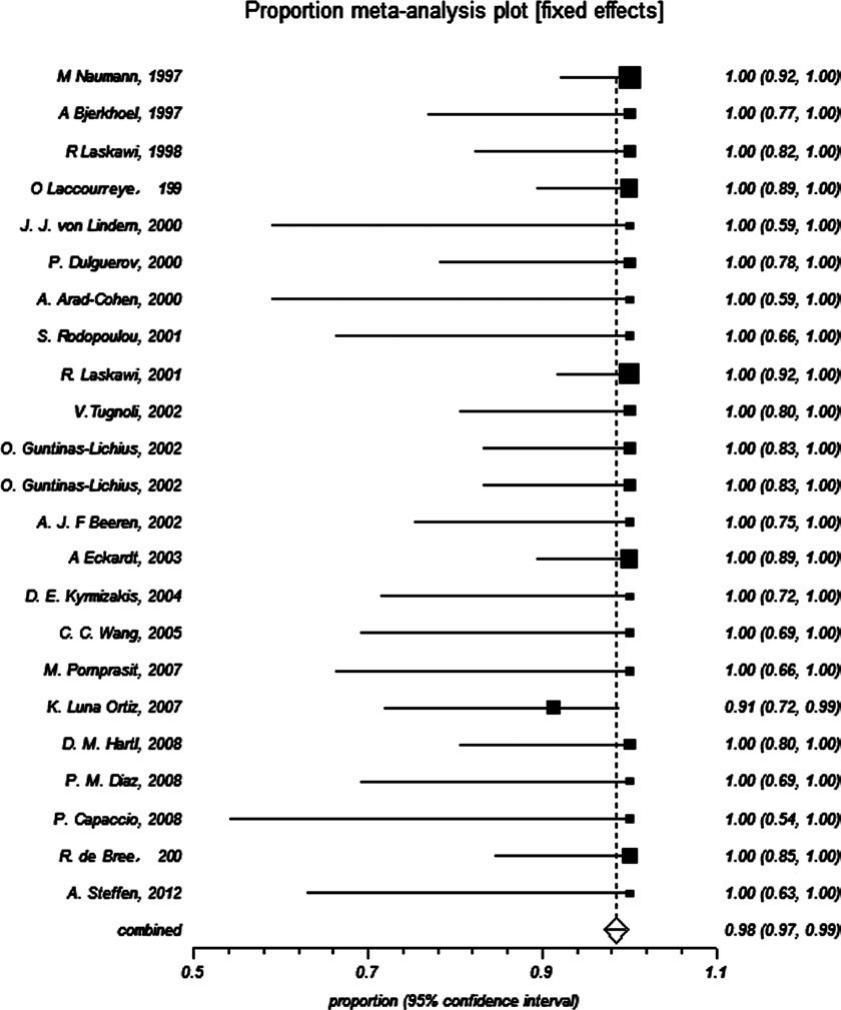
Effective rate of botulinum toxin type A in treatment of Frey’s syndrome (proportion meta-analysis).

All the eligible studies were involved in the efficacy of BTXA in treatment of FS. Only two patients had no response to the BTXA management in these 411 patients. Proportion meta-analysis suggested that I-squared (inconsistency) was equal to 0%, and thus the fixed effect model was used. The results of proportion meta-analysis showed that pooled proportion was equal to 0.984592 (95% CI = 0.970878–0.99402), which indicated that the effective rate of BTXA in treatment of FS was about 98%, and with 95% CI at least 97% (Fig.2).

### Current evidence on the safety of BTXA in treatment of FS

**Figure 3:**
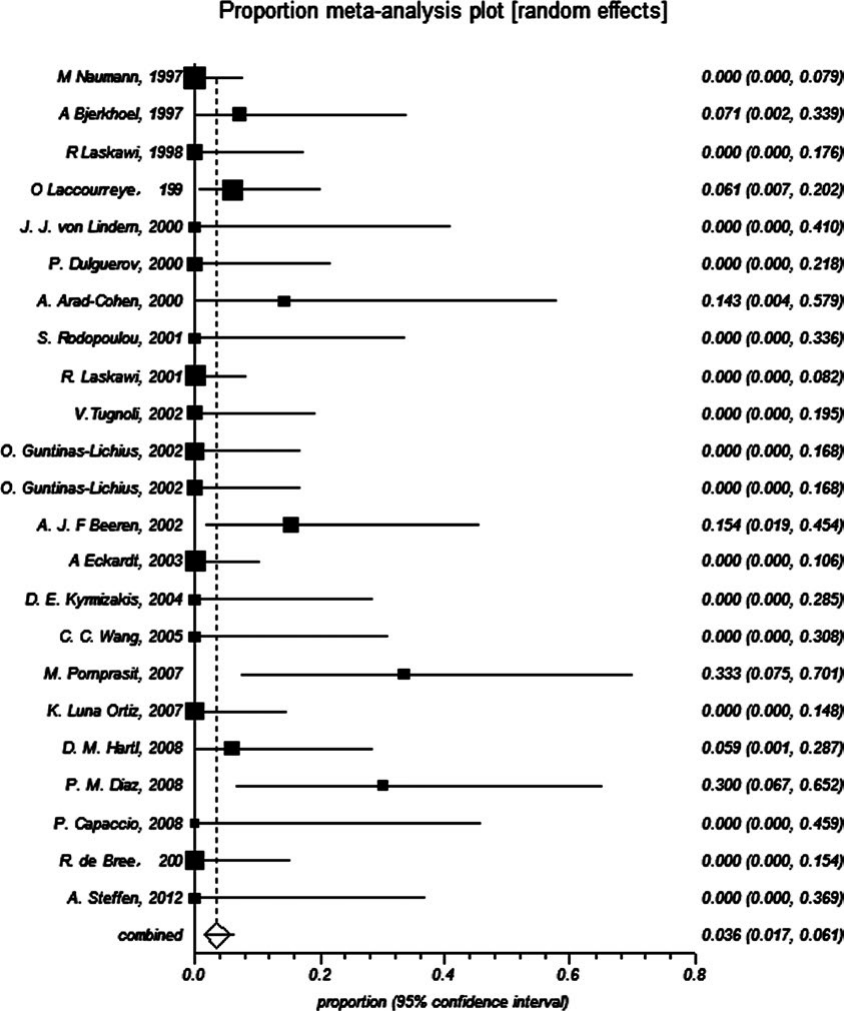
Incidence of complications regarding of botulinum toxin type A in treatment of Frey’s syndrome (proportion meta-analysis).

In all included patients, five patients suffered from dry mouth and eight patients suffered from transient muscular weakness or numbness, which completely recovered in 12 weeks (Table1). Proportion meta-analysis showed that I-squared (inconsistency) was equal to 33.1%, and thus random effect model was performed. The results of proportion meta-analysis suggested that pooled proportion was equal to 0.03602 (95% CI = 0.017151–0.061475), which indicated that the complication incidence of BTXA in treatment of FS was about 3.6%, and with 95% CI at most 6% (Fig.3). Besides, about 30 subjects suffered from weak or moderate pain after injections or at the time of injections (Table1).

## Discussion

Although the use of BTXA in the treatment of FS is becoming widely accepted as a better option to traditional therapy, only 23 case series studies, to date, have been reported to estimate this topic. Treatment strategies of these investigations included various courses of injection, injecting at diverse levels with different concentration botox, interjection distance, volume and dose. The methods of BTXA for treatment of FS in the included literature were listed in Tables1 and  2. Most of them seemed to agree that an interjection distance of 10 mm, a volume per injection point of 0.1 mL and 2.5 U Botox concentration (10 U Dysport ≈ 2.5 U Botox) was performed for patients with FS. In these existing studies, Dulguerov et al. and Guntins-Lichius et al. performed a concentration of Botox of 5 U/0.1 mL and 5 U in per injection site 38,40. The latter author declared that the higher dosage of BTXA might increase the symptom-free interval without a higher risk of complications (the mean duration of effect reached 20 months) 38. However, the former did not offer enough follow-up time of their patients 40. Unfortunately, Steffen and his colleagues performed a concentration of Botox of 5 U/0.1 mL in per injection site, which still failed to demonstrate this conclusion after 10 years later (mean duration of effect reached 7.8 months) 27. This situation might have resulted from lower volume per injection site (0.05 mL) than previous volume per injection site (0.1 mL), which indicated that 0.1 mL per injection sites might be a better option than 0.05 mL. The injection distance of 20 mm 25,34,36,41 produced similar mean duration of effect to 10 mm 24,35,37,44 indicating no significant associations between injection distance and mean duration of effect. The maximal dose used in per participants ranged from 30 to 380 U without serious side effects, suggesting that 380 U for patients with FS might be a safety dose. To sum up, this systematic review recommended the following treatment strategies: (1) the interjection distance varied from 10 to 20 mm; (2) the volume per injection point was 0.1 mL; (3) the Botox concentration ranged from 2.0 to 5.0 U/mL; and (4) the maximal dose was no more than 380 U per participants.

Strong evidence on the efficacy of BTXA in treatment of FS relies on reliable original data. Unfortunately, there is a shortage of high quality of evidence in this issue. Although the effective rate reached 98.5% for the treatment of BTXA in FS, it is of difficulty to identify whether all of the effects are produced by BTXA because of the deficiency of the randomized control group. Thus, the existing evidence regarding the effectiveness of BTXA in treatment of FS remains weak.

In these 23 studies, nearly all the mean duration of effect was more than 6 months except one study that did not report the details of follow-up time 33 and two articles had short follow-up time 40,42. Noticeable, 11 studies had more than 10 months of mean duration of effect, and the effect time even lasted more than 20 months without recurrence in some patients 25. Generally, BTXA injection is an effective treatment with a long-lasting effect for FS.

As for the recurrence of patients, most of investigators performed the treatment of BTXA again and the good results were reported at the same time. Noticeable, Remco de Bree and his colleagues claimed that the duration of effect increases after BTXA injections for patient who received repeated treatment 41. However, Guntinas-Lichius et al. declared that the first BTXA treatment did not influence the dose of BTXA needed for the renewed treatment after recurrence 38 and Tugnoli et al. found that no decrease in the efficacy of BTXA after renewed injections 26.

As for the safety of BTXA, only 13 patients suffered from complications, including five patients for dry mouth and eight patients for muscular weakness, which recovered completely in 3 months. Besides, there were several reports about the tolerable pain at the time of injection or after injection, but most of them disappeared in two weeks. Without an RCT, however, it is impossible to judge whether of all the complications were relevant with the BTXA. After all, all injections including saline injection also might produce the similar complications. In a short, this systematic review suggests that it is of safety and effectiveness to use BTXA in treatment for patients with FS.

Although this review has offer a systematical and scientific estimation for BTXA in the treatment of FS, several limitations in our study have to be noticeable. Firstly, literature retrievals were limited to those existing articles in English, which might miss the potentially eligible studies in other languages. Secondly, all eligible articles were case series studies; generally, prospective RCTs would be needed to produce credible and robust conclusions. Thirdly, the follow up time of several articles were short and No. of patients were small, which may also influence the robustness of results. Fourthly, the treatments of BTXA in FS were performed by various surgeons or clinicians, which might also produce bias. Besides, The OCEBM quality of evidence is level 4, which indicates that future studies might change the conclusions. Based on these limitations, results of promotion should be cautious.

Recently, Dessart claimed that the injection of BTXA is the first-line treatment of FS 45. However, Li and his colleagues declared that they were unable to establish the efficacy and safety for the treatment of FS because lacking of RCTs 46. As a matter of fact, observational studies – even case-series and anecdotes might sometimes provide definitive evidence 47,48. To this controversy, we offer our views with objective evidence as the results mentioned above. All systematic reviews regarding clinical treatments should link their conclusion with practice. BTXA was first used to treat FS in 1995 by Drobic and his colleagues 16,17. Although there were no RCTs to support its wide use, it did produce a few complications and meaningful benefits on the treatment of patients with FS in the past 20 years (Data were shown in Figs.2 and 3). Why there were no RCTs concerning this topic? We have to interpret this problem from the actual situations. First, BTXA is potentially effective to FS; second, BTXA treats patients with FS with few reported complications; third, patients are informed consent and all desiring for this treatment; fourth, no better methods are available instead of BTXA in treatment of FS until now. Besides, what drugs or other treatment methods would be used as control group? If we use the drug with serious complication or saline or worse treatment methods, it might violate medical ethics and produce unfavorable results for patients. Thus, BTXA has been widely used in clinical treatment without RCT evidence, which might be due to the limitations of clinical practice and medical ethics. In short, RCT is an ideal approach but difficult to perform on this topic.

## Conclusion

BTXA consistently improves the outcomes of patients with FS, even recurrent patients, with good efficacy and safety. However, owing to lack of strong controlled evidence to refute or support the usage of BTXA for treating patients with FS, future studies with well-designed inclusion criteria and multicenter RCTs are needed to give more credible conclusions, if possible.
